# Detecting DoS Attacks through Synthetic User Behavior with Long Short-Term Memory Network

**DOI:** 10.3390/s24123735

**Published:** 2024-06-08

**Authors:** Patrycja Nędza, Jerzy Domżał

**Affiliations:** Faculty of Computer Science, Electronics and Telecommunications, AGH University of Krakow, 30-059 Krakow, Poland; pnedza@student.agh.edu.pl

**Keywords:** Denial of Service, machine learning, behavioral telemetry, Long Short-Term Memory

## Abstract

With the escalation in the size and complexity of modern Denial of Service attacks, there is a need for research in the context of Machine Learning (ML) used in attack execution and defense against such attacks. This paper investigates the potential use of ML in generating behavioral telemetry data using Long Short-Term Memory network and spoofing requests for the analyzed traffic to look legitimate. For this research, a custom testing environment was built that listens for mouse and keyboard events and analyzes them accordingly. While the economic feasibility of this attack currently limits its immediate threat, advancements in technology could make it more cost-effective for attackers in the future. Therefore, proactive development of countermeasures remains essential to mitigate potential risks and stay ahead of evolving attack methods.

## 1. Introduction

Modern Denial of Service (DoS) attacks are becoming larger and more sophisticated every year, which imposes the need for robust defense mechanisms utilizing cutting-edge technologies [1,2,3,4]. With ML emerging as a promising tool in not only defense, but also attack execution, it is becoming necessary to explore its potential usage by attackers and to stay ahead of evolving threats. This study investigates the feasibility of leveraging ML for DoS attack detection by generating behavioral telemetry data using a Long Short-Term Memory (LSTM) network [5] and Bezier Curves, and crafting spoofing requests to make the analyzed traffic appear legitimate.

While previous research has explored ML-based approaches for DoS attack detection and mitigation, few studies have examined the potential of generating realistic attack traffic through ML itself. The authors of [6] present the machine learning techniques that can be implemented to enhance efficiency of Intrusion-Detection Systems (IDSs). For example, Average One Dependence Estimators (AODE) is presented as one of the recent improvements of the Naive Bayes algorithm. It is explained that the IDS using the AODE was proposed to detect different types of attacks with high accuracy. Among the other analyzed methods, the Random Forest, K-means or decision tree can be indicated. The authors conclude their review by stating that further work on reducing the false alarm rate and increasing the detection rate are desirable. The authors of [7] propose an IDS that can handle the entire packet information in a network with high efficiency using the hierarchical Long Short-Term Memory. This paper proves that the use of LSTM allows one to increase the detection rate of attacks significantly. However, there are still possibilities to successfully evade an ML-based IDS. In reference [8], an attack-resistance analysis of IDSs is provided. The authors show that, in existing systems, based on ML models, attackers can evade IDSs with up to 35.7% success rates. They also propose a new solution to counteract these weaknesses. Three defense mechanisms, model voting ensembling, ensembling adversarial training and query detection, are presented and it is shown that these methods can improve intrusion-detection rates to nearly 100% when considering most types of malicious traffic. Network intrusion-detection models are based on e.g., Multilayer Perceptron (MLP), Convolutional Neural Network (CNN) and LSTM. Another solution based on LSTM is presented in [9]. The authors propose an adversarial DBN-LSTM method to detect and protect against DDoS attacks in an SDN environment. The experiments conducted using the public dataset CICDDoS 2019 show that the proposed method allows for efficient detection of known DDoS attacks compared to other approaches. Taking into account the high effectiveness of solutions based on LSTM and their growing popularity, we decided to use this method in a new solution for simulating realistic DoS attacks.

This research contributes to the field by:

- Demonstrating the potential of LSTM networks to generate realistic behavioral telemetry data that can be used for DoS attack simulations.
- Evaluating the effectiveness of spoofing requests in masking attack traffic and challenging current detection methods.
- Analyzing the economic feasibility of ML-based DoS attacks and highlighting the need for proactive countermeasures despite current limitations.

Our findings aim to accelerate the development of machine learning-based DoS attack-detection systems by providing valuable insights into attack methodologies and potential blind spots in existing defenses. What is new is the use of solutions based on LSTM in this area.

## 2. Related Work

In reference [10], the authors propose an adversarial Deep Belief Network–Long Short-Term Memory (DBN-LSTM) method for detecting and defending against DDoS attacks. The key point of the system is the adversarial DBN-LSTM anomaly-detection method. It can generate several adversarial samples and build adversarial datasets using the generative adversarial network (GAN) model. Moreover, other proposals have been presented to improve the performance of the system:

- Implementation of DBN for data dimensionality reduction;
- Implementation of LSTM to extract sample timing features to detect IP flow records and identify adversarial DDoS attacks.

The simulation experiments show that the proposed method can effectively detect DDoS attacks and improve system sensitivity to adversarial attacks. In this paper, we propose some new techniques to protect systems against the ML-based DoS attacks.

The authors of [11] presented a conditional GAN-based intrusion-detection system against DDoS attacks on IoT networks. The core of the system is the mechanism which generates synthetic traffic mapping known patterns and completely new network discriminator networks to detect anomalies. The obtained results confirm that the generated dataset significantly improved the detection effectiveness. An important element of the entire solution is the mechanism based on deep learning classifiers. In our proposal, we use different ML-based models to detect specific DoS attacks.

In [12], an LSTM-based system was proposed to detect and prevent DDoS attacks in public cloud networks. The system was designed based on a signature-based attack-detection approach. The accuracy rate is on the level of 99.83% according to the CICDDoS2019 dataset. The simulation results confirm that the prevention part of the system obtained a performance as good as for the previous studies conducted with different DL algorithms on the same and different datasets. In our approach, we use the LSTM-based system with data generated by behavioral telemetry. This is unique for currently known studies.

The review of currently known deep learning-based approaches for detecting DDoS attacks is presented in [13]. The review classifies the papers into five main research areas:

- Types of DDoS attack-detection deep learning approaches;
- Methodologies, strengths, and weaknesses of existing deep learning approaches for DDoS attack detection;
- Benchmarked datasets and classes of attacks in used datasets;
- The preprocessing strategies, hyperparameter values, experimental setups, and performance metrics;
- The research gaps, and future directions.

Another review which presents different approaches to detecting DDoS attacks, using machine learning techniques is presented in [14]. Analyzed techniques, such as K-means, K-Nearest Neighbors, and Naive Bayes used in intrusion-detection systems and flow-based intrusion-detection systems were considered for review. In the review, the high-speed network accuracy-evaluation factors are highlighted. They provide a detailed DDoS attack taxonomy, and classify detection techniques. Several types of attacks were considered, e.g., Zero-Day Attacks, Reflection Attacks, DNS Amplification or SYN Flood.

In [15], a novel parallel model that integrates Convolutional Neural Network and Long Short-Term Memory was proposed. It allowed one to achieve higher accuracy than previous studies regarding detection of DoS attacks. By utilizing the NSL_KDD dataset, the presented model achieved an accuracy of 99.45% in detecting Denial of Service attacks.

In [16], the exploratory model of Multi-layer Perceptron (MLP) classifiers using deep neural network algorithms was proposed to improve the attack-detection system. The final outcome shown in the paper results in higher accuracy and precision by dealing with a huge dataset that was gathered. It was reported that the accuracy and precision was obtained at 98.85% and 92%, respectively.

The mechanism proposed in our paper has not been noticed in the analyzed literature presented in both review papers presented above.

## 3. Testing Environment

The testing environment simulates a simple login/register website utilizing JavaScript listeners for background data collection. Upon a user’s visit to the application, a continuous stream of requests to “telemetry.js” is initiated. This script captures both mouse position and keyboard key press timestamps, sending them to the server via POST requests. The pseudocode in Algorithm 1 outlines the implemented logic. The first section of the algorithm is responsible for collecting data from the keyboard, while the second part reads coordinates of mouse position. Finally the function SendTelemetryData sends the obtained data to two dedicated databases: Telemetry Database and Request Database.
**Algorithm 1** Telemetry Data Collection**upon** Document is fully loaded **do**     **on** KeyDown event on window **do**        timestamp← Current timestamp        Call SendTelemetryData with ‘keyboard’ and timestamp     **end on**     **on** MouseMove event on window **do**        timestamp← Current timestamp        mouseX← Mouse X coordinate from event        mouseY← Mouse Y coordinate from event        Call SendTelemetryData with ‘mouse’, timestamp, mouseX, mouseY     **end on**     **function** SendTelemetryData(eventType, timestamp, x, y)        Send POST request to ‘/track_telemetry’ with event data        **on** Response received **do**           Log response message        **end on**     **end function****end upon**

1. The Telemetry Database captures user interactions with the website, including:
  - Event ID (unique identifier),
  - IP address and source port,
  - Event type (keyboard or mouse),
  - Timestamp,
  - Mouse X and Y coordinates (applicable only for mouse events).
2. The Request Database logs information about individual requests made to the website, such as:
  - Request ID (unique identifier);
  - Telemetry data hash (linking request to interaction);
  - IP address and source port;
  - Timestamp (time of current request);
  - HTTP headers, status code, method, hostname, path;
  - Interaction start time (when was first GET request to critical endpoint sent).

To enhance security, user behavior data (telemetry) are collected only on critical pages like login and register. This allows us to focus verification efforts on areas with heightened risk of bot activity. Specifically, a unique telemetry hash is calculated after credentials are submitted via a POST request.

The hash-generation process, outlined in Algorithm 2, considers two key factors:

- Mouse movement: we capture a string representing mouse position points throughout the interaction;
- Keystroke cadence: the average time between key presses is calculated based on timestamps collected since the page loaded successfully.

The algorithm runs the SendRequestData function which for each POST action computes the hash of telemetry_str. After retrieving necessary data and initializing variables, entry data are analyzed, concerning whether they represent mouse or keyboard. For keyboard, time between key presses is additionally analyzed. Finally, computed hash and updated time strings are sent to the database.
**Algorithm 2** Telemetry Data Hash**function** SendRequestData     timestamp← Current datetime     resp← Request headers as dictionary     telemetry_hash← ‘N/A’     Initialize database cursor conn     **if** Request method is ‘POST’ **then**         Retrieve telemetry data between interaction start and current timestamp         data← Fetched data         **if** data is not empty **then**            Initialize telemetry_str, key_timestamps, total_time_diff, count            **for** each entry *d* in data **do**               **if** d[3] is ‘mouse’ **then**                   Append mouse coordinates to telemetry_str               **else if** d[3] is ‘keyboard’ **then**                   Add d[4] to key_timestamps               **end if**            **end for**            Calculate average time between key presses            Append average time to telemetry_str            Compute hash of telemetry_str         **end if**         Insert request data into the database    **end if**    Commit changes to the database    Close database connection**end function**

### Attack Execution

The attack begins by stealthily gathering crucial information about the target login page. This involves pinpointing the exact positions of input boxes and the submit button, as meticulously outlined in Algorithm 3.
**Algorithm 3** Get Initial Position of Element**function** GetInitialPosition(host, path, element_id, offset)      Initialize headless Chrome browser with fullscreen and SSL error ignore options      Navigate browser to “https://{host}{path}”      Find the element on the webpage by its ID      Get the screen dimensions using PyAutoGUI      Retrieve browser window position      Calculate the center coordinates of the element      Adjust coordinates to be within screen limits and apply offset      Quit the browser      **return** Screen coordinates (screen_x, screen_y)**end function**

The algorithm triggers the GetInitialPosition function, which contains several elements that are launched sequentially. The goal is to find coordinates of the elements placed on the web page.

Armed with this knowledge, the attack commences by sending a carefully crafted stream of POST requests. Each request bears a deceptive payload: meticulously crafted mouse position data, designed to mimic genuine human interaction. This process, detailed in Algorithm 4, aims to evade detection by blending seamlessly with typical user behavior.

The algorithm triggers the SendTelemetryRequest function, which constructs data as a JSON string, which is equipped with coordinates and host, session, and browser details. Finally the string is sent with path, data, and necessary headers.
**Algorithm 4** Send Telemetry Request**function** SendTelemetryRequest(event_type, timestamp, x (optional), y (optional))    Define path as ‘/track_telemetry’    Construct data as a JSON string with event_type, timestamp, x, and y    Define headers with necessary information including host, session, and browser details    Call send_post_request with path, data, and headers**end function**

Mouse positions are calculated, leveraging Bézier curves, widely used in computer graphics, game development, and image processing, to generate realistic mouse trajectories. These curves offer flexibility and smooth transitions, crucial for mimicking natural user behavior. It is important to understand that those curves can be exchanged with new ML models, such as “SapiAgent”, to generate even more human-like mouse trajectories [17]. However, for simplicity, we will operate on the Bézier curves. They are defined by Bernstein polynomials as shown in Equation (1) [18],

(1) BZ(t)=∑i=0nniti(1−t)n−iPi,0≤t≤1

where:

- *n*—the degree of the Bézier Curve (number of control points minus one);
- *t*—the parameter of the Curve (position along the curve ranging between 0 to 1).

We strategically choose three control points for each curve:

- Start point A;
- End point B;
- Three random points on the line connecting A and B, with a random perpendicular offset.

This point-selection process is elucidated in Algorithm 5.
**Algorithm 5** Generate Random Point on Line  1:**function** GenerateRandomPointOnLine(point_a, point_b, randomness)  2:    t← Random number between 0 and 1  3:    x←(1−t)×point_a.x+t×point_b.x  4:    y←(1−t)×point_a.y+t×point_b.y  5:    Calculate the directional difference dx, dy between point_b and point_a  6:    Determine perpendicular direction perp_dx, perp_dy  7:    Normalize the perpendicular direction  8:    Calculate random_distance based on randomness  9:    Adjust *x*, *y* coordinates by applying random_distance in the perpendicular direction10:    **return** *x*, *y*11:**end function**

By leveraging Equation (1), we numerically calculate the Bézier curve points, as outlined in Algorithm 6.
**Algorithm 6** Generate Bézier Curve Points  1:**function** GenerateBezierCurve(control_points, num_points)  2:    t← Create a list of num_points evenly spaced values between 0 and 1  3:    Initialize curve as a num_points×2 zero matrix  4:    n← Number of control points minus 1  5:    **for** i←0 to num_points−1 **do**  6:          **for** j←0 to *n* **do**  7:           Calculate Bernstein polynomial bernstein_poly  8:           curve[i]←curve[i]+bernstein_poly×control_points[j]  9:         **end for**10:    **end for**11:    **return** curve12:**end function**

**Figure 1:**
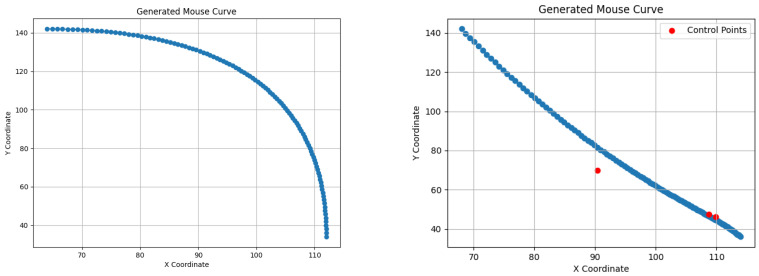
Bézier Curves generated (**left**) by ready python framework with two control points; (**right**) by calculating curve points with five control points.

To visualize the generated curves and enhance understanding, we present them in Figure 1.

Once the simulated mouse reaches its intended input field, the attack shifts its focus to simulating keyboard activity. This attack leverages the assumption that attackers can readily build a database of keyboard dynamics for their use. To simulate real user behavior, a simple keylogger was implemented. The code samples 128 random passwords from the “rockyou.txt” file [19], one at a time. For each password, it meticulously records every keystroke, capturing both the exact moment the key is pressed (key_down) and released (key_up). These precise timing data continue until the user presses “Enter”, signaling the completion of the typed word. Captured data are then stored in a comma-separated value (CSV) file. The structure of this database is outlined in Table 1.

Each row uniquely identifies a keystroke with a timestamp (recorded in milliseconds), and associates it with the typed word (using an internal Word_ID) and the corresponding ASCII code.

**Figure 2:**
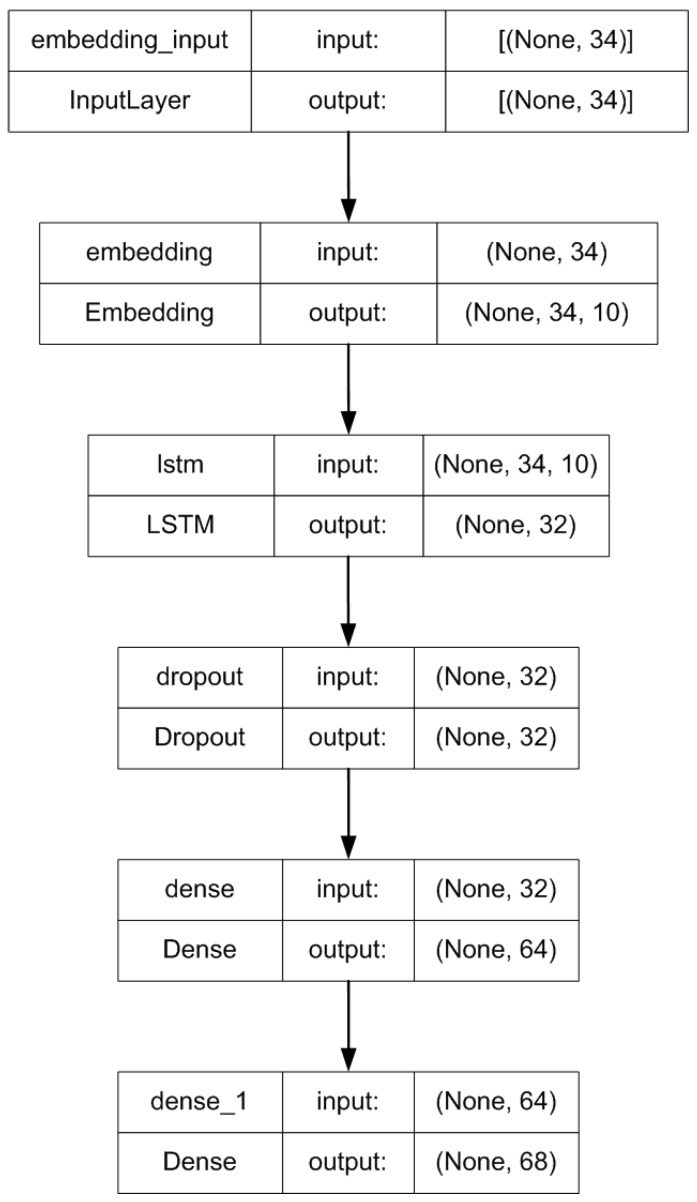
Model architecture.

**Figure 3:**
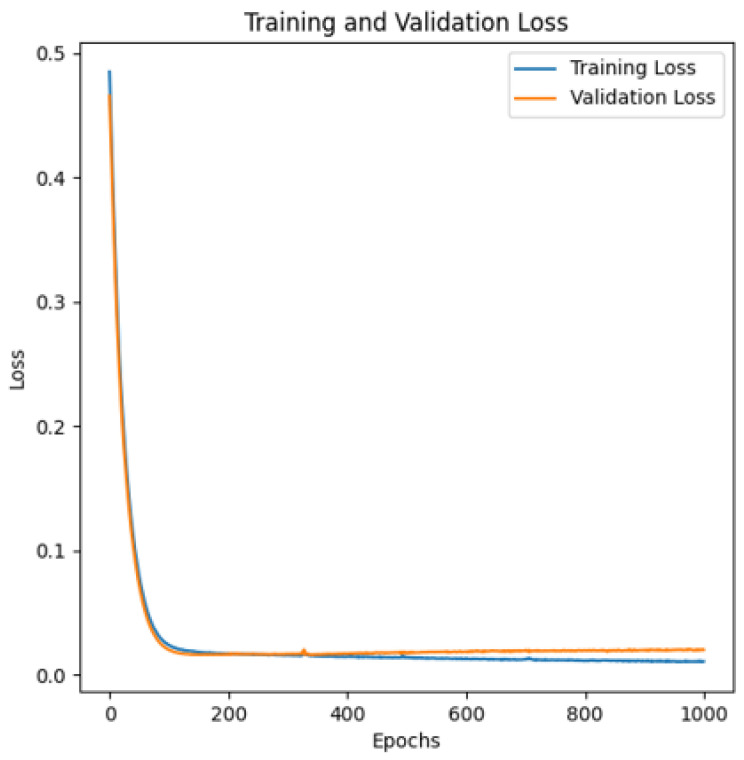
Training and Validation Loss of the model.

A Long Short-Term Memory (LSTM) model serves as the backbone for predicting the intervals between keystrokes [5]. This choice leverages the inherent sequential nature of keystroke timing data, allowing the model to capture and learn crucial temporal dependencies. While our problem was quite simple, we used one hidden layer. A dense layer with 10 nodes per layer was implemented. The dropout value was set to 20%. This value is widely accepted as the best compromise between preventing model overfitting and retaining model accuracy. A uniform distribution was used to choose initial weight values. Decay rate was set to the default value of 0.97. Figure 2 visually represents the model’s structure. The model loss is shown on Figure 3.

Specifically, the model uses 34 input features, representing the maximum password length from the generated database. This allows the model to understand the potential range of keystroke sequences. The LSTM layer then processes this information efficiently, extracting appropriate patterns and relationships from data. Finally, the output layer utilizes these learned patterns to predict the time a key should be held for, as well as the interval between the current and next keystroke.

Algorithm 7 precisely predicts key press and release timings, ensuring a natural typing cadence. These meticulously timed events are then transmitted to the server, further solidifying the illusion of a human presence.
**Algorithm 7** Type In Current Input Field**function** TypeInCurrentField(word)    **global** timestamp    times ← predict_hold_and_release_times(word)    **for** each time in times **do**         offset ← create time delta from time[0]         send_telemetry_request(“keyboard”, timestamp + offset)         increment timestamp by offset and additional time from time[1]    **end for****end function**

After successfully filling each input field, the attack seamlessly resumes its simulated mouse movements, guiding the cursor towards the submit button. Upon reaching this critical element, the attack culminates in the final submit button click. To maximize the likelihood of success, the attack relentlessly repeats this intricate sequence, relentlessly attempting passwords from the expansive “rockyou.txt” file. This unwavering persistence ensures that no potential password combination is overlooked.

## 4. Results

The attack was conducted on the self-written testing website with login/register functionality, running a JavaScript event listener in the background. It allowed us to collect behavioral data such as mouse position and timestamp of key press. Based on those data, a telemetry hash was calculated and stored in the server database, along with other information on the request such as the exact headers, status code, etc. All tests were performed on the localhost, using laptop with AMD Ryzen 5 4500U CPU and integrated GPU Radeon Graphics 2.38 GHz. The testing environment was equipped with 16 GB RAM and used the 64-bit Windows 10 operating system. As machine learning was involved, the chosen programming language was Python. It comes with available machine learning frameworks like tensorflow or keras, which were used in the process of making the keyboard dynamics model. For reading the .csv table, the Pandas framework was used. The testing application itself was created with the Flask framework. Data were stored on a MySQL Windows server using three separate databases. Information about registered users was stored in dedicated databases. The attack was based on the assumption the attacker could easily create a database for the keyboard dynamics used in the attack. For that reason, a simple key-logger was implemented, which created a .csv file on the output. Once the user visited the Flask application, a constant stream of requests to telemetry.js was made. There, the information about current mouse position and keyboard key press timestamps was collected and sent to the server via POST requests. Telemetry data were collected only on \login and \register pages. Those pages are critical from a security standpoint and need a special point of verification against bot activity. Code sampled 128 random passwords from the rockyou.txt file and recorded all key presses along with the key_up/key_down times until enter was clicked, indicating the end of the typed word. Logic was repeated for each word until all the data were collected and written into a .csv file.

We examined the request data, focusing on the following key columns:

- ID;
- Telemetry hash;
- IP address;
- Port;
- Timestamp.

To illustrate the differences introduced by behavioral telemetry, we present excerpts of request data from two sets of simulations:

1. Without Behavioral Telemetry: As shown in Table 2, requests lack unique telemetry hashes. Bot traffic resulted in generating likewise hashes, making it potentially easier to mark as fraudulent.
2. With Behavioral Telemetry: Table 3 demonstrates the presence of randomized telemetry hashes, indicating the traffic was more sophisticated in the way behavioral telemetry was used. These unique identifiers, generated based on user behavior simulated during the attack, add a layer of complexity and potentially enhance the attack’s ability to evade detection.

Executing the attack yielded several clues about its impact on the server. The first one was the frequent number of 500 Internal Server Errors being logged. These responses suggest the server struggled to handle the influx of requests, potentially overloading resources. Another suggestion is the “ConnectionResetError” seen at the peak of traffic received by the server. This error indicates the server actively terminated connections, likely as a defense mechanism.

**Figure 4:**
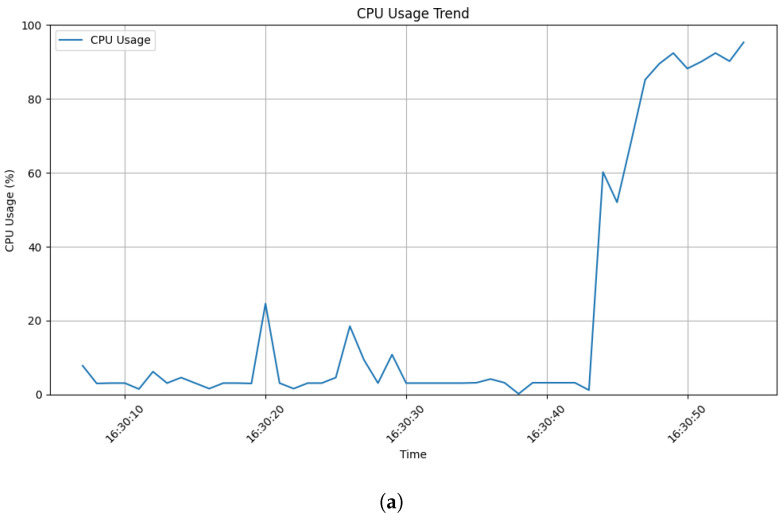

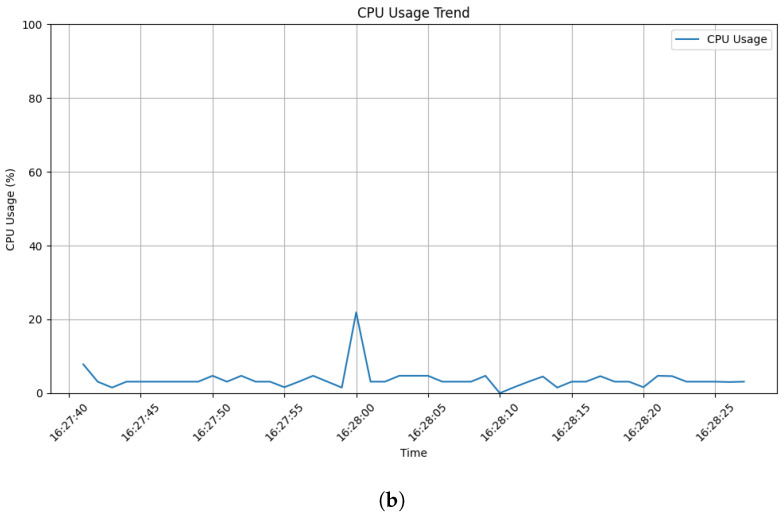
CPU usage trend: (**a**) during attack; (**b**) during legitimate user interaction.

To gauge the attack’s resource consumption, we instrumented the server with dedicated threads. One thread continuously recorded Central Processing Unit (CPU) usage at a specified interval, while the other tracked memory usage. Figure 4 reveals how the attack affected CPU utilization. Unlike legitimate traffic, which exhibits periods of lower usage (subfigure_b), CPU usage during the attack remained consistently high (subfigure_a). This indicates the server struggled to process the influx of requests, likely exceeding its capacity. Furthermore, the lack of clear data at the end of the attack period (subfigure_a) suggests that resources were saturated, preventing complete file writing. As a result, the recorded data had to be truncated.

**Figure 5:**
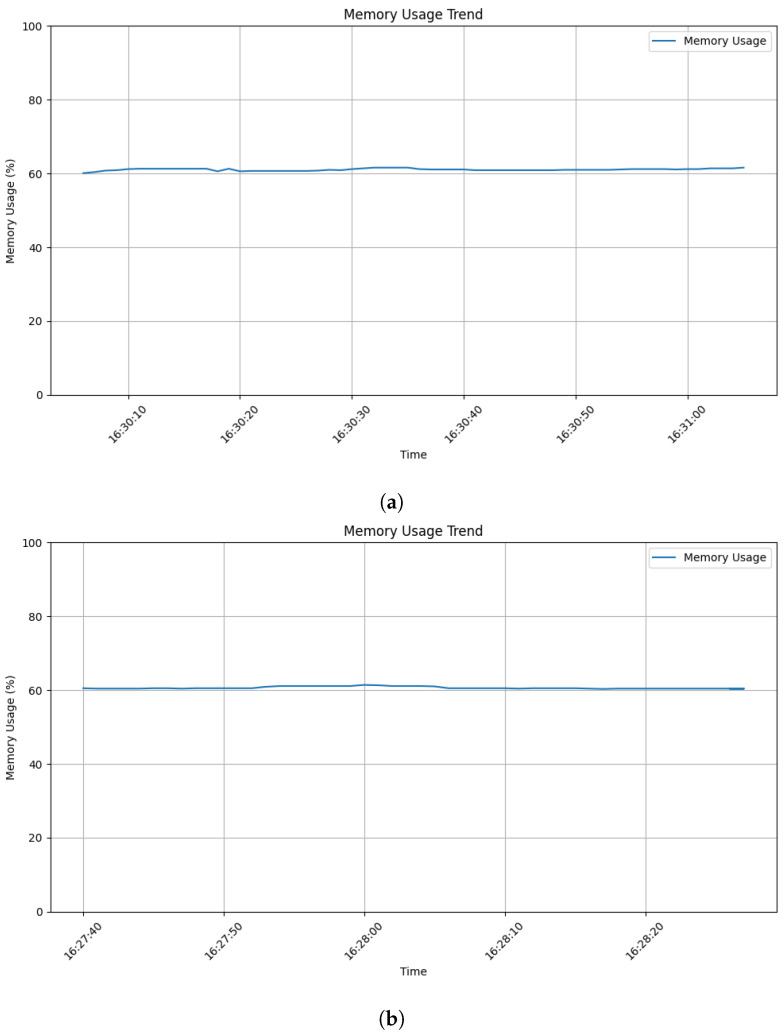
Memory usage trend: (**a**) during attack; (**b**) during legitimate user interaction.

While CPU usage offered clear insights, Figure 5 shows no significant differences in memory consumption on the web application server during the attack (subfigure_a) compared to normal use (subfigure_b). This suggests the attack primarily impacted CPU resources. However, we expect the changes to be reflected in storage utilization on the Windows MySQL server, which requires further investigation.

**Figure 6:**
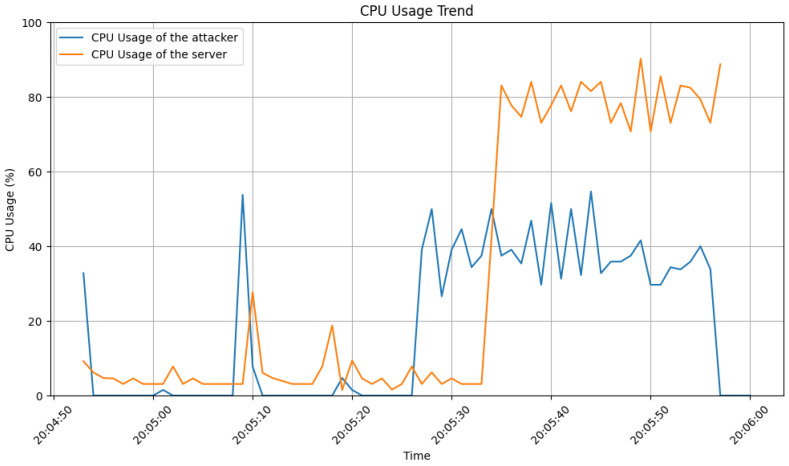
Attacker CPU usage compared to the server CPU usage.

Figure 6 compares the CPU usage of the attacker’s machine with the server throughout the attack. While the server experienced sustained high usage, the attacker’s CPU usage dropped to 0% just when the server shut down.

While the attack demonstrably impacted server resources, its efficiency from the attacker’s perspective is questionable. Many other DoS techniques offer a better “workload–result ratio”, meaning they achieve similar disruption with less resource investment. However, this advantage comes at the cost of easier detection. Table 4 illustrates a captured request sent during the attack. Generally, for the application itself, headers appear legitimate and do not differ from a typical request sent by a legitimate user.

It is important not to rely on timestamps collected by JavaScript, as they can be easily spoofed. Instead, similarly to the example presented in this paper, the timestamp is replaced on the back-end of the server once the POST request with telemetry data is received. Some systems may collect telemetry data for a period of time before sending the behavioral data to the server as a calculated hash to make it more difficult to spoof than straight x and y values. In such a scenario, it may be more difficult to review the data against spoofed values as hashes are non-reversible. Conversely, when logging all information, we can easily detect IP addresses, which were attempting to spoof the data, as shown in Table 5.

## 5. Conclusions and Future Work

The presented study analyzed a DoS attack using forged telemetry data, the aim of which was to imitate legitimate user behavior and avoid detection. Our analysis revealed:

- Deceptive Nature: The attack exploited the system’s dependence on telemetry data for user identification and resource allocation. This highlights the inherent weaknesses of trust-based security models in complex systems.
- Detection Challenges: The ability of the attack to blend into typical user behavior requires advanced detection methods that go beyond simple anomaly-based approaches.

However, while data collection is performed through controlled simulations, it may not fully capture the complexity of real-world attack scenarios. Additionally, the analysis focused on a specific type of attack, and the effectiveness of the scam may vary depending on the approach.

Our findings confirm previous research highlighting the increasing sophistication of DoS attacks and their increasing use of deceptive techniques. Moreover, the challenges of detecting deception attacks result in the need for advanced threat-detection methods that combine behavior analysis with anomaly detection.

The proposed solution can certainly be useful for detecting DoS attacks, but above all it shows the method that should be used in attack-detection systems. Today, the activities of malicious users are very complex and sophisticated. Attack-detection systems must have functionalities implemented to detect suspicious behavior, even though they increasingly imitate real users. Our solution shows directly how to strengthen protection against this type of attack. However, our results represent a first step in the development of this type of approach. In the future, we plan to undertake a deeper analysis of the possibilities of using the proposed solution. We will certainly optimize the solution’s operation by using modern machine learning models. It will be necessary to provide detailed comparative analyses with existing solutions.

## Figures and Tables

**Table 1:** Generated database structure.

Field Name	Field Type	Key Primary
Timestamp	Date-Time	True
Word_ID	Integer	False
ASCII_Code	Integer	False
Down_Time_MS	Float	False
Up_Time_MS	Float	False

**Table 2:** Part of the request data for simulation without behavioral telemetry spoofing used.

ID	Telemetry Hash	IP Address	Port	Timestamp
291	e3b0c44298fc1c149afbf4c8996fb92427…	192.168.1.13	64513	2023-11-16 18:19:02
294	e3b0c44298fc1c149afbf4c8996fb92427…	192.168.1.13	64513	2023-11-16 18:19:07
297	e3b0c44298fc1c149afbf4c8996fb92427…	192.168.1.13	64513	2023-11-16 18:19:35
300	e3b0c44298fc1c149afbf4c8996fb92427…	192.168.1.13	64513	2023-11-16 18:19:48
310	e3b0c44298fc1c149afbf4c8996fb92427…	192.168.1.13	64513	2023-11-16 18:22:43

**Table 3:** Part of the request data for simulation with behavioral telemetry spoofing used.

ID	Telemetry Hash	IP Address	Port	Timestamp
444	393e9cd505f0a7af71979ffb01e97468d…	192.168.1.13	53546	2023-11-16 18:56:23
446	4d373db4bafae80bddefd995a4d4a6d90…	192.168.1.13	53546	2023-11-16 18:56:27
448	bd970844de692ae66388c6ce161d0d41f…	192.168.1.13	53546	2023-11-16 18:56:30
454	25329f94ba7a792f5d4605cf94bd2240d…	192.168.1.13	53546	2023-11-16 18:58:53
456	a866e3a3fa5ec701b12f242dad5128f92…	192.168.1.13	53546	2023-11-16 18:58:56

**Table 4:** Example attack POST request headers received by the server.

Host	192.168.1.13
**Accept**	text/html,application/xhtml+xml, application/xml;q=0.9,image/avif,image/webp,image/apng,*/*;q=0.8, application/signed-exchange;v=b3;q=0.7
**Cookie**	session=.eJwlzLsKwyAARuFXkX8WU VND6guEDC0dsos0NhWsgpcuIe9eoeOBw3dgeUBDXCUT48QEEwMofKwu22f 1KZpSba7QB8jWx_XdKBEjuacvkVwORExaKX1RZL6tOClC2ne3GRhXzYUR9G B2gq05JyiFZeN71JsIfwz2o_r8txcqTh_vpMrnA.ZVZXxw.h1HjL5HRu_cm9I9MK _dJJnbRcqg
**Origin**	https://192.168.1.13
**Referer**	https://192.168.1.13/login
**Sec-Ch-Ua**	Google Chrome;v=“119”, “Chromium”;v=“119", “Not?A_Brand";v=“24"
**Connection**	keep-alive
**User-Agent**	Mozilla/5.0 (Windows NT 10.0; Win64; x64) AppleWebKit/537.36 (KHTML, like Gecko) Chrome/119.0.0.0 Safari/537.36
**Content-Type**	application/x-www-form-urlencoded
**Cache-Control**	max-age=0
**Content-Length**	30
**Sec-Fetch-Dest**	document
**Sec-Fetch-Mode**	navigate
**Sec-Fetch-Site**	same-origin
**Sec-Fetch-User**	?1
**Accept-Encoding**	gzip, deflate, br
**Accept-Language**	en-US,en;q=0.9
**Sec-Ch-Ua-Mobile**	?0
**Sec-Ch-Ua-Platform**	Windows
**Upgrade-Insecure-Requests**	1

**Table 5:** Fragment of the Telemetry table.

ID	IP Address	Port	Event Type	Timestamp	Mouse X	Mouse Y
92	192.168.1.13	54513	keyboard	2023-11-07 22:32:48		
93	192.168.1.13	54513	keyboard	2023-11-07 22:32:48		
94	192.168.1.13	54513	keyboard	2023-11-07 22:32:48		
95	192.168.1.13	54513	mouse	2023-11-07 22:32:49	144	66
96	192.168.1.13	54513	mouse	2023-11-07 22:32:49	154	77
97	192.168.1.13	54513	mouse	2023-11-07 22:32:49	172	86

## Data Availability

The original contributions presented in the study are included in the article, further inquiries can be directed to the corresponding author/s.

## Abbreviations

The following abbreviations are used in this manuscript:

DoS	Denial of Service
ML	Machine Learning
LSTM	Long Short-Term Memory
GAN	Generative Adversarial Network
IDS	Intrusion-Detection System
CSV	Comma-Separated Values
CPU	Central Processing Unit
